# Sedentary Behavior and Lower-Extremity Physical Function across the Lifespan of Adults with Multiple Sclerosis

**DOI:** 10.3390/ijerph191912466

**Published:** 2022-09-30

**Authors:** Brenda Jeng, Petra Šilić, Trinh L. T. Huynh, Robert W. Motl

**Affiliations:** 1 Department of Kinesiology and Nutrition, College of Applied Health Sciences, University of Illinois Chicago, Chicago, IL 60612, USA; 2 Department of Physical Therapy, School of Health Professions, The University of Alabama at Birmingham, Birmingham, AL 35223, USA

**Keywords:** multiple sclerosis, aging, lifespan, physical function, balance, gait, strength

## Abstract

One outcome of aging with multiple sclerosis (MS) involves the decline in physical function, including compromised balance, reduced walking speed, and lower-extremity strength. Sedentary behavior, the other end of the activity continuum, may be targeted for improvements in physical function among adults with MS, but less is known about the relationship between sedentary behavior and physical function with increasing age in MS. This study examined the associations between device-measured volume and pattern of sedentary behavior and physical function based on SPPB (Short Physical Performance Battery) of ambulatory persons with MS across the lifespan. We categorized participants (N = 216) into young (20–39 years), middle-aged (40–59 years), and older (60–79 years) age groups. Participants completed the SPPB during a single visit to the laboratory and wore an accelerometer for a 7-day period. The one-way analysis of variance indicated no differences in volume and pattern of sedentary behavior among the three age groups, except for average sedentary bout length. Spearman bivariate correlations indicated that pattern, but not volume, of sedentary behavior was associated with physical function in young, middle-aged, and older adults, and the magnitude of these associations varied by age group. Future interventions may reduce and break up prolonged periods of sedentary behavior for improvements in physical function and possibly other consequences in persons with MS.

## 1. Introduction

Multiple sclerosis (MS) is a prevalent, chronic neurological disease of the central nervous system, and the disease process manifests as demyelination and transection of axons as well as loss of neurons over time [1]. The onset of MS typically occurs in early and middle adulthood [2], but there is a growing prevalence of older adults with MS [3]. This indicates that MS is a disease that occurs across the adult lifespan, and supports the examination of differences in outcomes and health behaviors across the lifespan of adult age groups with MS.

One outcome of aging with MS involves the decline in lower-extremity physical function, including compromised balance, reduced walking speed and lower-extremity strength [4,5]. This has been captured using the Short Physical Performance Battery (SPPB) [6]. The SPPB has been commonly applied in clinical and research settings for measuring declines in physical function with aging in the general population [7,8], as it provides a comprehensive assessment of lower-extremity physical function in adults across the lifespan [9]. To date, there is no evidence of declines in physical function with aging in MS, but this pattern is present in adults of the general population [9].

Physical activity has been identified as a health behavior for possibly improving physical function in older adults with MS [10], as time spent in light and moderate-to-vigorous physical activity has been associated with better physical function as measured by the SPPB in older persons with MS [11]. Data from a randomized controlled trial (RCT) further indicated that a 6-month, home-based exercise training program improved physical function in 307 older adults with MS [12]. Another RCT reported a moderate effect of a 12-week home-based exercise training program on physical function in older adults with MS [13]. Physical activity may be an efficacious and feasible approach for improving physical function and other health-related outcomes in persons with MS across the lifespan, yet persons with MS are not sufficiently active for accruing health benefits [14], and this worsens with aging [15].

One approach for promoting physical activity involves reducing and/or breaking up sedentary behavior and replacing it with physical activity. Indeed, sedentary behavior is on the other end of the activity continuum, and there is an increasing interest in sedentary behavior in MS [16]. Sedentary behavior is defined as any waking activity performed sitting or lying that does not increase energy expenditure above 1.5 metabolic equivalents of task [17]. Sedentary behavior is prevalent among persons with MS [18], and the volume of sedentary behavior (minutes per day) has been associated with older age in MS [19]. This indicates that there is a large reservoir of sedentary behavior in MS, particularly among older adults with MS, that can be targeted for physical activity promotion and improvements in physical function among adults with MS.

The provision of approaches for reducing or breaking up sedentary behavior in MS requires evidence of association between sedentary behavior and physical function, yet, to date, there is limited research examining both the volume and pattern of sedentary behavior as potential correlates of physical function in MS across the lifespan. One study examined the association between volume of sedentary behavior and physical function in 40 older adults with MS and reported no significant association between the time spent in sedentary behavior and summary SPPB scores [11]. One other study examined the associations between metrics of volume and pattern of sedentary behavior and SPPB summary and component scores (i.e., gait speed, lower-extremity strength) of 40 older adults with MS and 40 controls matched by age and sex [10]. The results indicated significant moderate-to-large associations (*r* = −0.41–0.56) among time spent in sedentary behavior, sedentary bout length, long sedentary bout length, and summary SPPB scores as well as significant moderate-to-large associations (*r* = −0.33–0.62) among time spent in sedentary behavior, long sedentary bout length, and component SPPB scores in older adults with MS. Such initial results support further investigation of sedentary behavior across the lifespan in MS, as the research suffers from limitations including the relatively small sample sizes, restricted age range (i.e., older adults), and lack of focus on sedentary behavior across the lifespan in MS.

The current study examined the associations between device-measured volume and pattern of sedentary behavior and physical function based on SPPB summary and component scores of persons with MS across young, middle-aged, and older adults with MS. We expected that the volume and pattern of sedentary behavior would be associated with SPPB summary and component scores across age groups with the strongest associations in the older group. If we identify a metric of volume and/or pattern of sedentary behavior as a correlate of physical function, this may inform the design of behavioral interventions that target reductions and/or break-up of sedentary behavior as an approach for improving physical function in persons with MS of a specific age group.

## 2. Materials and Methods

### 2.1. Participants

Persons with MS were recruited through word of mouth, flyers in the community, advertisement through the local MS chapter, neurology clinics, and a therapeutic recreation facility. Participants who expressed interest contacted the laboratory via email or phone. Participants then underwent a phone screening for eligibility based on the following criteria: (1) MS diagnosis; (2) no relapse within the last 30 days; (3) age between 20–79 years; (4) ability to walk with or without assistive devices; and (5) willingness to complete the study protocol. Participants were categorized into young (20–39 years), middle-aged (40–59 years), and older (60–79 years) age groups.

### 2.2. Procedures

The study protocol was approved by the University Institutional Review Board. All participants provided written consent prior to data collection. Participants completed questionnaires regarding demographic and clinical characteristics and underwent an assessment of physical function during a single visit to the laboratory. Upon completion of the visit, participants were then instructed to wear an accelerometer for a 7-day period for a measure of sedentary behavior. Participants were remunerated for their time.

### 2.3. Measures

#### 2.3.1. Demographic and Clinical Characteristics

Participants completed a questionnaire related to demographic and clinical characteristics (i.e., age, sex, MS type, disease duration). Participants also self-reported their disability status using the Patient Determined Disease Steps (PDDS) [20].

#### 2.3.2. Sedentary Behavior

The ActiGraph model GT3X+ accelerometer provided a device measurement of sedentary behavior. The accelerometer was initialized to collect raw data (g force) at a sampling rate of 100Hz. The device was secured in a pouch on an elastic belt worn around the waist above the non-dominant hip during waking hours, except while showering, bathing, and swimming, for 7 days. The participants were instructed to record the date and duration of wear time for the accelerometer, and this daily log was inspected for verifying the days of wear time estimates from the ActiLife software during data processing. The accelerometer data were downloaded using the ActiLife software, reintegrated into activity counts in one-minute epochs with the low frequency extension, and then scored for time spent in sedentary behavior (<100 counts/min) per day. We considered a day as valid if there was a minimum of 10 hours of total wear time without continuous zeros exceeding 30 min, and participants with 1 or more valid days of data were included in the analyses. Outcomes of interest included (1) total sedentary time spent per day, (2) average number of breaks in sedentary time per day, defined as at least 2 min where the accelerometer registers ≥100 counts/min following a sedentary bout, (3) average sedentary bout length where a bout is a period of consecutive minutes were the accelerometer registers <100 counts/min, (4) average number of long sedentary bouts (>30 min) per day, and (5) average total time spent in long sedentary bouts expressed in minutes per day.

#### 2.3.3. Physical Function

The SPPB was used to assess physical function, including standing balance, gait speed, and lower-extremity strength [6,21]. For standing balance, participants were instructed to maintain upright posture for 10 seconds per assessment while standing with various stances: feet side-by-side, semi-tandem, and tandem. The balance assessments were administered in that order, whereby participants who were able to maintain balance with a given stance progressed to the subsequent, more challenging stance. For gait speed, participants walked a four-meter course at normal pace. For lower-extremity strength, participants were instructed to rise from a chair five times as quickly and safely as possible without the use of arms for support. Prior to undertaking the assessment, participants were first asked to attempt and complete a single practice of sit-and-rise as stated in the SPPB protocol. Component scores for each assessment and a summary score aggregating the standing balance, gait speed, and lower-extremity strength scores were calculated as per the standard SPPB protocol. Each component score ranged from 0 (inability to complete assessment) to 4 (highest level of performance). Component scores from the standing balance, gait speed, and lower-extremity strength assessments were then summed for a summary score, ranging from 0 to 12; higher scores indicated better physical function of the lower extremities.

### 2.4. Data Analysis

All data were analyzed using SPSS, Version 28. Descriptive statistics of the overall sample and per age group were provided as mean (SD), numbers (n) and percentages (%), and median (IQR) unless otherwise noted. We performed one-way analysis of variance (ANOVA) and chi-square tests to examine demographic and clinical characteristics, metrics of volume and pattern of sedentary behavior, and SPPB summary and component scores among the three age groups. We categorized the sample into disease duration groups by decade (i.e., 1–10 years, 11–20 years, and 21+ years) and performed one-way ANOVA to examine sedentary behavior and SPPB scores among the three disease duration groups. We further performed one-way analysis of covariance (ANCOVA) that examined sedentary behavior and SPPB summary and component scores by age groups and by disease duration groups while controlling for comorbidities. We reported partial eta-squared for describing the main effects and interaction from the ANOVA, and interpreted the values based on guidelines for small, medium, and large effects of 0.01, 0.06, and 0.14, respectively [22]. We conducted Spearman rank-order bivariate correlations among metrics of the volume and pattern of sedentary behavior and summary and component SPPB scores of the overall sample and per age group of persons with MS. The magnitude of effect sizes was interpreted as small, medium, and large based on values of 0.1, 0.3, and 0.5, respectively [22].

## 3. Results

### 3.1. Sample Characteristics

Demographic and clinical characteristics of the overall sample and across age groups are provided in Table 1. The sample (N = 216) consisted predominantly of persons who were female (76%), Caucasian (65%), and presented with relapsing-remitting MS (88%). Mean age of the sample was 49.6 (13.3 SD) years, and mean disease duration was 13.0 (9.6 SD) years. The sample had a median PDDS score of 1 indicating mild disability. There were statistically significant differences in age (*p* < 0.001), race (*p* = 0.001), and disease duration (*p* < 0.001) among the three age groups.

### 3.2. Descriptive Statistics for Sedentary Behavior and Physical Function

Descriptive statistics for volume and pattern of device-measured sedentary behavior and SPPB summary and component scores of the overall sample of persons with MS and per age group are provided in Table 2. Descriptive statistics for sedentary behavior and SPPB scores across disease duration groups are provided in Supplementary Table S1. The overall sample spent 497.7 (91.8) minutes in sedentary time per day. The average number of breaks in sedentary time per day was 5.5 (2.0). The average sedentary bout length was 44.2 (7.4) minutes. The total number of long sedentary bouts was 4.8 (2.0), and the average total time spent in such long sedentary bouts was 222.3 (108.7) minutes per day. Mean SPPB summary score was 10.3 (2.4), mean balance component score was 3.4 (1.0), mean gait component score was 3.6 (0.9), and mean strength component score was 3.3 (1.2). The volume and pattern of sedentary behavior of our sample of persons with MS were somewhat inconsistent with values previously reported in persons with MS as our sample engaged in less sedentary behavior than previously reported [19,23]. However, SPPB scores among the older adult age group were comparable with previous research in older adults with MS [4,5].

The one-way ANOVA indicated that there were no differences in volume and pattern of sedentary behavior among the three age groups, except for average sedentary bout length, which was longest among older adults compared with young adults (*F* = 4.412, *p* = 0.013). The results further indicated that older persons demonstrated worse physical function based on lower summary scores (*F* = 6.463, *p* = 0.002) as well as balance (*F* = 5.244, *p* = 0.006) and strength (*F* = 5.341, *p* = 0.005) component scores compared with young adults. There were no significant differences in the gait component scores among the groups (*p* = 0.077). Of note, these observations were consistent even after controlling for race. Similarly, the one-way ANOVA indicated no differences in volume and pattern of sedentary behavior among the three disease duration groups. Moreover, SPPB summary (*F* = 6.075, *p* = 0.003), balance (*F* = 6.935, *p* = 0.001), and strength (*F* = 3.495, *p* = 0.032) scores were significantly different among the three disease duration groups, whereas gait scores were not different.

The one-way ANCOVA indicated no differences in volume and pattern of sedentary behavior across the age groups and across the disease duration groups once we controlled for comorbidities. However, the results indicated a significant difference in SPPB summary scores (*F* = 3.917, *p* = 0.021, η^2^ = 0.038) as well as balance component scores (*F* = 4.523, *p* = 0.012, η^2^ = 0.043) but not gait or strength component scores.

### 3.3. Bivariate Correlations between Sedentary Behavior and Physical Function

Bivariate correlation coefficients between sedentary behavior variables and SPPB summary and component scores for the overall sample of persons with MS are provided in Table 3. The pattern, but not volume, of sedentary behavior was associated with SPPB summary and component scores. SPPB summary score was negatively associated with the average number of breaks in sedentary time per day (*ρ* = −0.190, *p* < 0.05), the average sedentary bout length (*ρ* = −0.312, *p* < 0.01), the total number of long sedentary bouts (*ρ* = −0.197, *p* < 0.01), and the average total time spent in those sedentary bouts (*ρ* = −0.210, *p* < 0.01), but not with total sedentary time per day (*ρ* = −0.076, *p* > 0.05). SPPB balance and gait component scores were associated with average sedentary bout length (*ρ* = −0.244, *p* < 0.01 and *ρ* = −0.169, *p* < 0.05, respectively). SPPB strength component score was associated with the average number of breaks in sedentary time per day (*ρ* = −0.193, *p* < 0.01), the average sedentary bout length (*ρ* = -0.234, *p* < 0.01), the total number of long sedentary bouts (*ρ* = −0.183, *p* < 0.05), and the average total time spent in those sedentary bouts (*ρ* = −0.193, *p* < 0.01), but not with total sedentary time per day (*ρ* = −0.092, *p* > 0.05). Overall, there was a weak, but significant, negative correlation between physical function scores and sedentary behavior pattern.

Bivariate correlation coefficients between sedentary behavior variables and SPPB summary and component scores across age groups are provided in Table 4. Among younger persons with MS (20–39 years), SPPB summary score was associated with sedentary behavior patterns (range of *ρ* between −0.354 and −0.350, *p* < 0.05), but not volume (*ρ* = −0.289, *p* > 0.05). SPPB balance, gait, and strength component scores were not associated with sedentary behavior volume or pattern.

Among middle-aged persons with MS (40–59 years), pattern, but not volume, of sedentary behavior was significantly associated with SPPB summary or component scores. Average sedentary bout length was associated with SPPB summary (*ρ* = −0.252, *p* < 0.05) and balance component (*ρ* = −0.249, *p* < 0.05) scores.

Among the older adult age group (60–79 years), SPPB summary scores were associated with the average number of breaks in sedentary time per day (*ρ* = −0.274, *p* < 0.05), the average sedentary bout length (*ρ* = −0.330, *p* < 0.05), the total number of long sedentary bouts (*ρ* = −0.346, *p* < 0.05), and the average total time spent in those sedentary bouts (*ρ* = −0.284, *p* < 0.05), but not with total sedentary time per day (*ρ* = −0.128, *p* > 0.05). SPPB balance component score was not significantly associated with volume or pattern of sedentary behavior. SPPB gait component score was associated with average sedentary bout length (*ρ* = −0.349, *p* < 0.01). Moreover, the SPPB strength component score was associated with the average number of breaks in sedentary time per day (*ρ* = −0.342, *p* < 0.05), average sedentary bout length (*ρ* = −0.325, *p* < 0.05), number of long sedentary bouts (*ρ* = −0.370, *p* < 0.01), and average total time spent in long sedentary bouts per day (*ρ* = −0.330, *p* < 0.05).

## 4. Discussion

This study examined volume and pattern of device-measured sedentary behavior as potential correlates of physical function based on SPPB scores across the lifespan in MS. Older adults with MS had the longer sedentary bout length and demonstrated worse physical function overall and by component (i.e., balance, lower-extremity strength) compared with young adults with MS. The bivariate correlation analyses indicated that pattern of sedentary behavior was associated with SPPB summary and component scores in middle-aged and older adults but only summary scores in young adults. This suggests that future efforts may consider the design of interventions that reduce and break up prolonged durations of sedentary behavior in persons with MS of specific age groups.

We examined device-measured volume and pattern of sedentary behavior in a relatively large sample of persons with MS across a wide range of age (20–79 years) and disease duration (1–48 years). Previous research identified age as a significant factor of both volume and pattern of sedentary behavior in MS [19]. Our results are somewhat consistent, in that only sedentary bout length rather than total time spent in sedentary behavior differed between young and older adults with MS even after controlling for race. The majority of research on physical function and sedentary behavior is conducted in White persons with MS, but there is evidence that the disease can manifest differently in other races such as Black persons with MS. Preliminary evidence suggests that Black persons with MS may have a worse disease course compared with White persons with MS. Based on the results of this study and previous research [11,15], future research efforts should focus on the development of interventions that both break up prolonged sedentary bouts and increase physical activity in persons with MS. Although sedentary behavior and physical activity (i.e., moderate-to-vigorous physical activity) are on the activity continuum, greater engagement in sedentary behavior does not necessarily suggest less participation in physical activity. Overall, pattern, but not volume, of sedentary behavior differ by age in persons with MS.

Furthermore, it is not surprising that young adults with MS had the highest scores of overall physical function compared with older counterparts. Previous research in walking performance, a proxy of physical function, across the lifespan has reported greater declines in physical function with increasing age [24]. Age-related factors such as severity of disease-related symptoms or the presence of comorbidities may have contributed to this difference in physical function across the age groups of MS [2,25,26]. When we categorized the sample by disease duration rather than age, the results were somewhat similar, in that physical function differed among the three groups. Of note, the age range of persons with shorter disease duration was larger than those with longer disease duration, and persons with longer disease duration were of older age. This is not surprising given that the growing prevalence of older adults with MS, and age and disease duration might have similar or unique influences on physical function. It is expected that age and disease duration are confounded. Moreover, our results indicated that the number of comorbidities can influence physical function (i.e., lower-extremity strength) in persons with MS. Older adults with MS had the most comorbidities, which might explain the reduced lower-extremity strength associated with aging. Interestingly, sedentary behavior was not different across the lifespan when accounting for disease duration or comorbidities, suggesting that sedentary behavior may not play as great of a role as physical activity does in physical function.

Previous research has used self-reported and device-measured volume of sedentary behavior in MS; however, device-measured patterns such as breaks in sedentary bouts or length of sedentary bouts can provide a better understanding of sedentary behavior and its outcomes in MS. The novelty of the current study was the inclusion of metrics regarding the pattern of sedentary behavior as potential correlates of physical function; the results indicated that in the overall sample of persons with MS, pattern rather than volume of sedentary behavior was related to physical function, mainly lower-extremity strength, in that persons who engage in more and longer sedentary bouts have worse overall physical function. However, when we categorized persons with MS by age, the magnitude of these relationships between metrics of sedentary behavior and physical function were stronger. These results are consistent with previous research, in that pattern of sedentary behavior may be a new target for management of disease-related outcomes in MS [16]. The findings suggest that future efforts may target increasing the number of breaks from sedentary behavior and decreasing time spent in sedentary bouts rather than reducing total time spent in sedentary behavior per day for improvements in physical function. Based on the results of this study on sedentary behavior and previous research on physical activity [10,11], future interventions may target reducing/breaking up sedentary behavior as well as increasing physical activity to improve physical function. Another potential avenue of future research may be the examination of the optimum number and length of sedentary and exercise bouts for improvements in physical function in persons with MS. Developing better patterns of sedentary behavior and increasing physical activity would not only improve physical function but may also reduce the risk of all-cause mortality as reported in adults of the general population [27,28,29].

The analyses indicated that only the pattern of sedentary behavior was related to summary scores in young, middle-aged, and older adults with MS and identified specific metrics of sedentary behavior as correlates of SPPB summary scores for each age group. Moreover, there were moderate associations between pattern of sedentary behavior and different SPPB component scores in middle-aged and older adults. For example, middle-aged adults who engaged in longer sedentary bouts had worse balance, whereas older adults with MS who were more sedentary based on all metrics of the pattern (i.e., higher number of breaks in sedentary time, longer sedentary bout length, more long sedentary bouts (>30 min), longer total time spent in sedentary bouts) demonstrated reduced strength. Older adults with MS who engaged in longer sedentary bouts further demonstrated impaired gait. However, there was no specific component score that was significantly related to patterns of sedentary behavior in young adults with MS despite the moderate-to-strong associations between summary scores and pattern. Overall, these novel findings suggest that certain metrics of sedentary behavior and physical function are related in each age group, and researchers have conducted feasibility studies that target reductions in sedentary behavior in sub-populations of MS such as persons of mild-to-moderate disability and Black/African Americans [30,31]. However, physical function has not been the main outcome of interest in these trials. Future research is warranted to examine the effects of breaking up sedentary behavior on physical function in MS.

There are some limitations to consider when interpreting these results. A limitation was the lack of a control, non-MS comparison group. Furthermore, although we had a relatively large sample of persons with MS across the lifespan, our sample had mild disability based on PDDS scores, which may have limited the variability of SPPB scores observed in this study. One possible interpretation is that the sample consisted of persons who were able to walk with or without assistive devices and excluded persons with more severe disability status. Another limitation was the cross-sectional design of this study, limiting inferences regarding causality between sedentary behavior and physical function of persons with MS. Furthermore, race did not matter when examining the relationship between sedentary behavior and physical function in our sample of persons with MS. We recruited participants from the southeast region of the United States, and this explains the diversity of our sample (i.e., White, Black, Hispanic); however, the focus of recruitment for the current study was based on age, not race. Future research with larger, inclusive samples may examine how the relationship between sedentary behavior and physical function changes over time. We further did not include data on factors that could affect sedentary behavior or physical function such as presence and/or severity of disease-related symptoms. Future efforts may examine, for example, the role of fatigue and fatiguability in the associations among physical activity, sedentary behavior, and physical function among older adults with MS.

## 5. Conclusions

Sedentary behavior is a relatively understudied behavior among persons with MS. The existing research has used self-reported measures of sedentary behavior, rather than devices such as accelerometers, and the self-reports only include volume, not pattern, of sedentary behavior. This study examined device-measured volume and pattern of sedentary behavior as possible correlates of physical function in persons with MS across the lifespan. Our findings suggest that pattern of sedentary behavior (i.e., sedentary bout length) and physical function differs significantly by age in MS. Pattern, not volume, of sedentary behavior was related to physical function overall and by component (i.e., balance but primarily lower-extremity strength) in the overall sample of persons with MS. However, we identified specific metrics of sedentary behavior as potential targets of physical function among young, middle-aged, and older adults with MS. Future interventions may break up the pattern of sedentary behavior for improvements in physical function and possibly other consequences in these age groups of MS.

## Figures and Tables

**Table 1:** Demographic and clinical characteristics of the overall sample and subsamples per age group of persons with multiple sclerosis (MS).

Characteristic	Overall (N = 216)	20–39 Years (N = 59)	40–59 Years (N = 93)	60–79 Years (N = 64)	*p*-Value
Age (years [SD])	49.6 (13.3)	32.9 (4.7)	49.3 (5.6)	65.5 (4.4)	<0.001
Sex (N, %)					0.882
*Female*	163, 76%	45, 76%	69, 74%	49, 77%	
*Male*	52, 24%	13, 22%	24, 26%	15, 23%	
Race (N, %)					0.001
*Caucasian*	140, 65%	27, 46%	59, 63%	54, 84%	
*Black/African American*	67, 31%	26, 44%	31, 33%	10, 16%	
*Other*	8, 4%	5, 9%	3, 3%	0, 0%	
MS Type (N, %)					0.184
*Relapsing remitting*	190, 88%	52, 88%	85, 91%	53, 83%	
*Progressive*	19, 9%	3, 5%	7, 8%	9, 14%	
*Unknown*	7, 3%	4, 7%	1, 1%	2, 3%	
Disease Duration (years [SD])	13.0 (9.6)	5.8 (4.9)	12.0 (7.5)	21.2 (9.5)	<0.001
PDDS score (0–8)	1 (3)	1 (3)	1 (3)	3 (3.5)	0.084

*Note:* PDDS = Patient Determined Disease Steps scale. PDDS score is presented as median (IQR).

**Table 2:** Overall volume and pattern of sedentary behavior and scores for Short Physical Performance Battery (SPPB) for the overall sample and across age groups of persons with multiple sclerosis.

Variable	Overall (N = 211)	20–39 Years (N = 57)	40–59 Years (N = 91)	60–79 Years (N = 63)	*p-*Value
Sedentary Behavior					
*Total sedentary time/day (min)*	497.7 (91.8)	490.9 (103.4)	507.4 (85.2)	489.7 (90.3)	0.449
*Average number of breaks in sedentary time per day (N)*	5.5 (2.0)	5.2 (2.3)	5.8 (1.9)	5.5 (1.8)	0.236
*Average sedentary bout length (min)*	44.2 (7.4)	41.8 (8.5)	44.5 (7.0)	46.0 (6.6)	0.013
*Number of long sedentary bouts (N)*	4.8 (2.0)	4.5 (2.3)	5.1 (1.9)	4.7 (1.9)	0.287
*Average total time spent in long sedentary bouts per day (min)*	222.8 (108.7)	200.4 (109.1)	230.8 (103.3)	230.9 (115.0)	0.238
SPPB					
*Summary (0–12)*	10.3 (2.4)	11.1 (2.1)	10.4 (2.3)	9.5 (2.8)	0.002
*Balance (0–4)*	3.4 (1.0)	3.7 (0.8)	3.5 (1.0)	3.1 (1.0)	0.006
*Gait (0–4)*	3.6 (0.9)	3.7 (0.8)	3.6 (0.8)	3.4 (1.1)	0.077
*Strength (0–4)*	3.3 (1.2)	3.7 (0.8)	3.3 (1.1)	3.0 (1.4)	0.005

*Note:* Values are presented as mean (SD).

**Table 3:** Spearman rank-order bivariate correlations between volume and pattern of sedentary behavior and summary and component scores of the Short Physical Performance Battery (SPPB) for the overall sample of persons with multiple sclerosis.

Variable	Overall (N = 202)			
	Summary	Balance	Gait	Strength
Total sedentary time/day (min)	−0.076	−0.036	−0.028	−0.092
Average number of breaks in sedentary time per day (N)	−0.190 *	−0.078	−0.105	−0.193 **
Average sedentary bout length (min)	−0.312 **	−0.244 **	−0.169 *	−0.234 **
Number of long sedentary bouts (N)	−0.197 **	−0.109	−0.109	−0.183 *
Average total time spent in long sedentary bouts per day (min)	−0.210 **	−0.112	−0.099	−0.193 **

*Note:* * *p* < 0.05; ** *p* < 0.01.

**Table 4:** Spearman rank-order bivariate correlations between volume and pattern of sedentary behavior and summary and component scores for the Short Physical Performance Battery (SPPB) across age groups of persons with multiple sclerosis.

Variable	20–39 Years (N = 51)				40–59 Years (N = 88)				60–79 Years (N = 63)			
	Summary	Balance	Gait	Strength	Summary	Balance	Gait	Strength	Summary	Balance	Gait	Strength
Total sedentary time/day (min)	−0.289	−0.240	−0.172	−0.242	0.020	−0.065	0.081	0.091	−0.128	0.054	−0.111	−0.249
Average number of breaks in sedentary time per day (N)	−0.350 *	−0.245	−0.187	−0.281	−0.080	−0.045	−0.028	−0.059	−0.274 *	−0.009	−0.190	−0.342 *
Average sedentary bout length (min)	−0.354 *	−0.229	−0.075	−0.149	−0.252 *	−0.249 *	−0.087	−0.172	−0.330 *	−0.133	−0.349 **	−0.325 *
Number of long sedentary bouts (N)	−0.276	−0.212	−0.113	−0.196	−0.089	−0.070	−0.035	−0.061	−0.346 *	−0.094	−0.259	−0.370 **
Average total time spent in long sedentary bouts per day	−0.351 *	−0.242	−0.123	−0.207	−0.096	−0.084	−0.012	−0.059	−0.284 *	−0.045	−0.205	−0.330 *

*Note:* * *p* < 0.05; ** *p* < 0.01.

## Data Availability

The data presented in this study may be made available upon request from the corresponding author. The data are not publicly available in accordance with funding requirements and participant privacy.

## Supplementary Materials

The following supporting information can be downloaded at: https://www.mdpi.com/article/10.3390/ijerph191912466/s1, Table S1: Overall volume and pattern of sedentary behavior and scores for Short Physical Performance Battery (SPPB) across disease duration groups of persons with multiple sclerosis
