# Supplementary material for: Sedentary Behavior and Lower-Extremity Physical Function across the Lifespan of Adults with Multiple Sclerosis
Source: Int J Environ Res Public Health. 2022 Sep 30;19(19):12466. doi: 10.3390/ijerph191912466 (PMC9566509; doi:10.3390/ijerph191912466)
Supplement: Supplementary file 1 [file ijerph-19-12466-s001.zip › ijerph-1920595-supplementary.pdf]

Table S1. Overall volume and pattern of sedentary behavior and scores for Short Physical Performance Battery (SPPB) across disease duration groups of persons with multiple sclerosis.

Variable	1–10 years (n = 98)	11–20 years (n = 71)	21+ years (n = 42)	*p*-value
Sedentary Behavior				
Total sedentary time/day (min)	504.8 (94.1)	501.0 (99.7)	476.9 (69.3)	0.275
Average number of breaks in sedentary time per day (N)	5.6 (2.1)	5.5 (2.0)	5.3 (1.5)	0.614
Average sedentary bout length (min)	43.7 (8.3)	44.8 (7.5)	44.6 (5.0)	0.627
Number of long sedentary bouts (N)	4.9 (2.2)	5.0 (2.0)	4.4 (1.6)	0.283
Average total time spent in long sedentary bouts per day (min)	226.7 (112.3)	22.7.9 (119.5)	205.7 (79.0)	0.547
SPPB				
Summary (0–12)	10.9 (2.0)	10.1 (2.9)	9.5 (2.6)	0.003
Balance (0–4)	3.6 (0.8)	3.4 (0.9)	3.0 (1.1)	0.001
Gait (0–4)	3.7 (0.7)	3.5 (1.1)	3.4 (0.9)	0.054
Strength (0–4)	3.6 (1.0)	3.2 (1.3)	3.0 (1.3)	0.032

Note: Values are presented as mean (SD).
